# Severe hypocalcemia following a single injection of denosumab in a patient with renal impairment

**DOI:** 10.3109/21556660.2012.668504

**Published:** 2012-02-21

**Authors:** Draupadi B. Talreja

**Affiliations:** Department of Medicine, David Geffen School of Medicine at the University of California, Los Angeles, CAUSA

**Keywords:** Denosumab, Hypocalcemia, Renal impairment, Safety

## Abstract

Monitoring renal function and adjusting dosing for patients with impaired renal function are not required with denosumab (60 mg every 6 months). However, these patients have an increased risk for developing hypocalcemia. This case report describes a patient with renal impairment who developed severe hypocalcemia after receiving denosumab.

## Introduction

A new antiresorptive therapy, denosumab (Prolia, Amgen Inc., Thousand Oaks, CA, USA; 60 mg every 6 months), is approved in the United States and Europe for the treatment of women with postmenopausal osteoporosis who are at high risk for fracture^1,2^. Approval in this setting was based on the Fracture Reduction Evaluation of Denosumab in Osteoporosis Every 6 months (FREEDOM) trial, which randomized women aged 60–90 years with postmenopausal osteoporosis (*N* = 7868; T-score <−2.5 but ≥−4.0) to receive denosumab (60 mg every 6 months) or placebo for 36 months. The trial showed that denosumab significantly reduced the risk of fracture (vertebral, *p* < 0.001; nonvertebral, *p* = 0.01)^2^. In the oncology setting, denosumab (Xgeva, Amgen Inc., Thousand Oaks, CA, USA; 120 mg every 4 weeks) is approved in the United States and Europe for use in patients with bone metastases from solid tumors, but not in patients with multiple myeloma^3^.

Denosumab is a fully human monoclonal antibody against receptor activator of nuclear factor κB ligand (RANKL), a key mediator of bone remodeling through the inhibition of osteoclast activity. Subset analyses of the FREEDOM trial showed that denosumab (60 mg every 6 months) was not associated with an increase in adverse events among patients with severe renal impairment (*n* = 73; creatinine clearance [CrCl] 15–29 mL/min) or impaired renal function (*n* = 2817; CrCl 30–59 mL/min) compared with those with normal renal function (*n* = 4911; CrCl ≥ 60 mL/min)^4^. Thus, the label for denosumab (60 mg every 6 months) does not require monitoring renal function prior to administration and only states that patients with CrCl < 30 mL/min or receiving dialysis are at increased risk for hypocalcemia^1^.

Bisphosphonates, another class of antiresorptive agent approved for the treatment of postmenopausal osteoporosis, are not indicated for patients with severe renal impairment (i.e., CrCl < 30–35 mL/min)^5–8^. Because denosumab has no such limitations and has been shown to be effective for the treatment of postmenopausal osteoporosis, it is considered a viable alternative for this patient population. Moreover, because denosumab is not contraindicated in patients with renal impairment, no dose adjustments based on renal function are available. However, patients with renal impairment who receive denosumab have an increased risk for the development of hypocalcemia^1^. Although no cases of symptomatic hypocalcemia were reported in the FREEDOM trial or in a trial of denosumab (60 mg every 6 months) compared with alendronate in postmenopausal women with osteoporosis^2^, the denosumab label states that severe hypocalcemia can occur in patients receiving denosumab^1^. Furthermore, because of an imbalance in the number of serious infections and dermatologic adverse events in the FREEDOM trial and the increased risk for developing hypocalcemia, the US Food and Drug Administration required a risk evaluation and mitigation strategy (REMS) for denosumab^9^. The REMS includes a medication guide that lists hypocalcemia as a side-effect and warns that this condition is often asymptomatic, but does not suggest routine monitoring of calcium levels^9^.

Untreated hypocalcemia may lead to chronic conditions such as cataract formation, prolonged QT interval, hypotension, congestive heart failure, seizures, or dementia. To minimize the risk for developing hypocalcemia, it is suggested that patients receiving antiresorptive therapy also receive daily calcium and vitamin D supplements. The denosumab label calls for concomitant calcium (1000 mg) and vitamin D (at least 400 mg), but monitoring of calcium levels prior to or during therapy is not required. Furthermore, although the denosumab label cautions that patients with severe renal impairment (i.e., CrCl <30 mL/min or receiving dialysis) are at risk for hypocalcemia, the only guidance provided by the label for use in this patient population is to supplement with calcium and vitamin D and to *consider* monitoring calcium levels^1^.

## Case report

The importance of monitoring calcium levels in patients with renal impairment is highlighted by an individual case of a 68-year-old woman with renal impairment who developed severe hypocalcemia after receiving a single 60-mg dose of denosumab. Patient comorbidities included chronic obstructive pulmonary disease, hypertension, osteoporosis, depression, rheumatoid arthritis, polycystic kidney disease, and chronic renal insufficiency. The patient was not able to tolerate alendronate, an oral bisphosphonate prescribed by her private medical doctor, which was discontinued on April 19, 2010, by Dr T. If an oral agent is not well-tolerated, both intravenous and subcutaneous routes of administration for bone supportive care agents are feasible options. Denosumab (60 mg every 6 months) was chosen over the intravenous bisphosphonate zoledronic acid because of the patient’s poor renal function. On October 13, 2010, denosumab was given per label to this patient with chronic renal insufficiency and no symptoms of hypocalcemia. Calcium (8.9 mg/dL) and serum creatinine (2.7 mg/dL) levels were last checked on June 2, 2010, 4 months prior to receiving denosumab. Other relevant laboratory values from this date included albumin (3.8 g/dL), alkaline phosphatase (47 U/L), total bilirubin (0.3 mg/dL), BUN (48 mg/dL), glucose (105 mg/dL), sodium (140 µg/L), potassium (4.7 µg/L), and chloride (109 µg/L).

Eleven days after denosumab administration (October 24, 2010), the patient presented at the hospital with fever and chills for 1–2 days, productive yellow cough with clear lungs and no shortness of breath, swelling, generalized pain and tenderness, mild confusion, and dyskinesia (i.e., twitching throughout the body). The patient was admitted to the hospital and was diagnosed with severe hypocalcemia (blood calcium 6.7 mg/dL). Thyroid hormone T4 (4.2 µg/dL), thyroid stimulating hormone (TSH, 0.726 µIU/mL), and vitamin D 1,25-dihydroxy (48 ng/mL) levels were normal, but parathyroid hormone (PTH) was high (409 pg/mL). Other relevant laboratory values included serum creatinine (2.23 mg/dL), albumin (2.1 g/dL), alkaline phosphatase (41 U/L), BUN (27 mg/dL), glucose (94 mg/dL), sodium (141 µg/L), potassium (4.5 µg/L), and chloride (116 µg/L). Blood calcium levels remained low (7.2 mg/dL) through October 28, 2010. With treatment (intravenous calcium gluconate, increased oral calcium, and continued vitamin D supplementation), the patient’s blood calcium returned to near normal (8.3 mg/dL) 3 weeks later (November 16, 2010).

## Discussion

Antiresorptive therapies inhibit bone resorption, which can reduce serum calcium levels in both normal and hypercalcemic individuals. Normal serum calcium levels are influenced by the effect of vitamin D 1,25-dihydroxy and PTH on calcium absorption, urinary calcium excretion, and bone remodeling activity in the skeleton (primary reservoir of calcium in the body)^10^. Therefore, antiresorptive therapy-mediated inhibition of bone resorption can lead to lower serum calcium levels and secondary hyperparathyroidism^10^, which could contribute to hypocalcemia, especially in individuals deficient in serum vitamin D or PTH^11,12^. Finally, renal insufficiency can lead to impaired conversion of vitamin D to its active metabolite (vitamin D 1,25-dihydroxy) and also may be a contributing factor to hypocalcemia^13^.

Hypocalcemia has not been reported with antiresorptive therapies (i.e., bisphosphonates or denosumab) in clinical studies of osteoporosis^2,14^. However, hypocalcemia has been reported in clinical trials of patients with cancer receiving antiresorptive therapies for metastatic bone disease. In patients receiving bisphosphonates (oral or intravenous) in this setting, the incidence of grade 3/4 hypocalcemia typically is not reported because of its low frequency and similar incidence with placebo^15^. Indeed, hypocalcemia was either not reported or was reported as an uncommon adverse event in clinical trials with bisphosphonates (i.e., clodronate, ibandronate, pamidronate, and zoledronic acid [ZOL])^15–29^. In contrast with the bisphosphonate trials, results from recent phase III clinical trials in patients with advanced cancer reported more frequent hypocalcemia with denosumab versus ZOL (5.5 vs. 3.4%, respectively, *p* < 0.05, in patients with breast cancer^30^; 10.8 vs. 5.8%, respectively, *p* = not reported, in patients with solid tumors or multiple myeloma^31^; 13 vs. 6%, respectively, *p* < 0.0001, in prostate cancer^32^). Furthermore, severe hypocalcemia was reported more often in patients receiving denosumab compared with ZOL (3.1% for denosumab vs. 1.3% for ZOL, *p* = not reported)^3^.

This case suggests that guidelines recommending a modified dosing schedule of denosumab (with corresponding modified dose vials) may be beneficial for patients with renal impairment, not because of nephrotoxic effects of the drug, but to avoid severe hypocalcemia. Furthermore, the case highlights the importance of monitoring calcium levels and renal function before and during denosumab therapy in patients with multiple comorbidities. Indeed, without monitoring renal function, how is a clinician to determine if a patient is at increased risk for developing hypocalcemia? Patients benefit from careful monitoring regardless of the route of administration (i.e., intravenous or subcutaneous) of antiresorptive therapies, and renal monitoring has positive benefits, particularly for patients with multiple comorbidities.

## Transparency

### Declaration of financial/other relationships

The author is on the speaker list for Warner Chilcott.

## References

[1] Prolia (denosumab) [package insert]. Thousand Oaks, CA: Amgen Inc., 2011

[2] Cummings SR, San Martin J, McClung MR, et al Denosumab for prevention of fractures in postmenopausal women with osteoporosis. N Engl J Med 2009;361:756-65 19671655 10.1056/NEJMoa0809493

[3] Xgeva (denosumab) [package insert]. Thousand Oaks, CA: Amgen Inc., 2010

[4] Jamal SA, Ljunggren O, Stehman-Breen C, et al Effects of denosumab on fracture and bone mineral density by level of kidney function. J Bone Miner Res 2011;26:1829-35 21491487 10.1002/jbmr.403

[5] Reclast (zoledronic acid) [package insert]. East Hanover, NJ: Novartis Pharmaceuticals Corporation, 2010

[6] Actonel (risedronate) [package insert]. Rockaway, NJ: Warner Chilcott (US), LLC, 2011

[7] Boniva (ibandronate) [package insert]. South San Francisco, CA: Genentech, Inc., 2011

[8] Fosamax (alendronate) [package insert]. Whitehouse Station, NJ: Merck & Co., Inc., 2011

[9] US Department of Health and Human Services. Risk evaluation and mitigation strategy (REMS) BL 125320 Prolia (denosumab). Available at: http://www.fda.gov/downloads/Drugs/DrugSafety/PostmarketDrugSafetyInformationforPatientsandProviders/UCM214383.pdf. Accessed October 31, 2011

[10] Gulley JL, Wu S, Arlen PM, et al Persistent hypocalcemia induced by zoledronic acid in a patient with androgen-independent prostate cancer and extensive bone metastases. Clin Genitourin Cancer 2007;5:403-5 17956715 10.3816/CGC.2007.n.025

[11] Breen TL, Shane E Prolonged hypocalcemia after treatment with zoledronic acid in a patient with prostate cancer and vitamin D deficiency. J Clin Oncol 2004;22:1531-2 15084635 10.1200/JCO.2004.99.013

[12] Navarro M, Lopez R, Alana M, et al Tonic-clonic seizure as the presentation symptom of severe hypocalcemia secondary to zoledronic acid administration. J Palliat Med 2007;10:1226-7 18095793 10.1089/jpm.2007.0113

[13] Mishra A Symptomatic hypocalcemia following intravenous administration of zoledronic acid in a breast cancer patient. J Postgrad Med 2008;54:237 18626181 10.4103/0022-3859.41815

[14] Black DM, Delmas PD, Eastell R, et al Once-yearly zoledronic acid for treatment of postmenopausal osteoporosis. N Engl J Med 2007;356:1809-22 17476007 10.1056/NEJMoa067312

[15] Pavlakis N, Schmidt RL, Stockler M Bisphosphonates for breast cancer. Cochrane Database Syst Rev 2005:CD003474 10.1002/14651858.CD003474.pub2 16034900

[16] Body JJ, Diel IJ, Lichinitser MR, et al Intravenous ibandronate reduces the incidence of skeletal complications in patients with breast cancer and bone metastases. Ann Oncol 2003;14:1399-405 12954579 10.1093/annonc/mdg367

[17] Body JJ, Diel IJ, Lichinitzer M, et al Oral ibandronate reduces the risk of skeletal complications in breast cancer patients with metastatic bone disease: results from two randomised, placebo-controlled phase III studies. Br J Cancer 2004;90:1133-7 15026791 10.1038/sj.bjc.6601663 PMC2409647

[18] Conte P, Guarneri V Safety of intravenous and oral bisphosphonates and compliance with dosing regimens. Oncologist 2004;9:28-37 15459427 10.1634/theoncologist.9-90004-28

[19] Hultborn R, Gundersen S, Ryden S, et al Efficacy of pamidronate in breast cancer with bone metastases: a randomized, double-blind placebo-controlled multicenter study. Anticancer Res 1999;19:3383-92 10629624

[20] Kanis JA, McCloskey EV, Powles T, et al A high incidence of vertebral fracture in women with breast cancer. Br J Cancer 1999;79:1179-81 10098755 10.1038/sj.bjc.6690188 PMC2362233

[21] Kohno N, Aogi K, Minami H, et al Zoledronic acid significantly reduces skeletal complications compared with placebo in Japanese women with bone metastases from breast cancer: a randomized, placebo-controlled trial. J Clin Oncol 2005;23:3314-21 15738536 10.1200/JCO.2005.05.116

[22] Kristensen B, Ejlertsen B, Groenvold M, et al Oral clodronate in breast cancer patients with bone metastases: a randomized study. J Intern Med 1999;246:67-74 10447227 10.1046/j.1365-2796.1999.00507.x

[23] Lipton A, Theriault RL, Hortobagyi GN, et al Pamidronate prevents skeletal complications and is effective palliative treatment in women with breast carcinoma and osteolytic bone metastases: long term follow-up of two randomized, placebo-controlled trials. Cancer 2000;88:1082-90 10699899 10.1002/(sici)1097-0142(20000301)88:5<1082::aid-cncr20>3.0.co;2-z

[24] Rosen LS, Gordon D, Kaminski M, et al Long-term efficacy and safety of zoledronic acid compared with pamidronate disodium in the treatment of skeletal complications in patients with advanced multiple myeloma or breast carcinoma: a randomized, double-blind, multicenter, comparative trial. Cancer 2003;98:1735-44 14534891 10.1002/cncr.11701

[25] Rosen LS, Gordon D, Tchekmedyian NS, et al Long-term efficacy and safety of zoledronic acid in the treatment of skeletal metastases in patients with nonsmall cell lung carcinoma and other solid tumors: a randomized, phase III, double-blind, placebo-controlled trial. Cancer 2004;100:2613-21 15197804 10.1002/cncr.20308

[26] Rosen LS, Gordon D, Tchekmedyian S, et al Zoledronic acid versus placebo in the treatment of skeletal metastases in patients with lung cancer and other solid tumors: a phase III, double-blind, randomized trial—the Zoledronic Acid Lung Cancer and Other Solid Tumors Study Group. J Clin Oncol 2003;21:3150-7 12915606 10.1200/JCO.2003.04.105

[27] Saad F, Gleason DM, Murray R, et al A randomized, placebo-controlled trial of zoledronic acid in patients with hormone-refractory metastatic prostate carcinoma. J Natl Cancer Inst 2002;94:1458-68 12359855 10.1093/jnci/94.19.1458

[28] Saad F, Gleason DM, Murray R, et al Long-term efficacy of zoledronic acid for the prevention of skeletal complications in patients with metastatic hormone-refractory prostate cancer. J Natl Cancer Inst 2004;96:879-82 15173273 10.1093/jnci/djh141

[29] Tripathy D, Lichinitzer M, Lazarev A, et al Oral ibandronate for the treatment of metastatic bone disease in breast cancer: efficacy and safety results from a randomized, double-blind, placebo-controlled trial. Ann Oncol 2004;15:743-50 15111341 10.1093/annonc/mdh173

[30] Stopeck AT, Lipton A, Body JJ, et al Denosumab compared with zoledronic acid for the treatment of bone metastases in patients with advanced breast cancer: a randomized, double-blind study. J Clin Oncol 2010;28:5132-9 21060033 10.1200/JCO.2010.29.7101

[31] Henry DH, Costa L, Goldwasser F, et al Randomized, double-blind study of denosumab versus zoledronic acid in the treatment of bone metastases in patients with advanced cancer (excluding breast and prostate cancer) or multiple myeloma. J Clin Oncol 2011;29:1125-32 21343556 10.1200/JCO.2010.31.3304

[32] Fizazi K, Carducci M, Smith M, et al Denosumab versus zoledronic acid for treatment of bone metastases in men with castration-resistant prostate cancer: a randomised, double-blind study. Lancet 2011;377:813-22 21353695 10.1016/S0140-6736(10)62344-6 PMC3090685

